# Utilization of Instrumentation in Swallowing Assessment of Surgical Patients during COVID-19

**DOI:** 10.3390/life13071471

**Published:** 2023-06-29

**Authors:** Heather Warner, Jennifer M. Coutinho, Nwanmegha Young

**Affiliations:** 1 Department of Surgery, Section of Otolaryngology, Yale School of Medicine, New Haven, CT 06510, USA; 2 Department of Communication Disorders, Southern Connecticut State University, New Haven, CT 06515, USA; 3 Department of Rehabilitation, Yale New Haven Hospital, New Haven, CT 06510, USA

**Keywords:** dysphagia, deglutition, surgical, swallowing assessment, Fiberoptic Endoscopic evaluation of swallowing, COVID-19

## Abstract

**Simple Summary:**

COVID-19 has significantly altered healthcare delivery worldwide. The pandemic presented significant challenges for the assessment of dysphagia in the acute care setting, particularly for surgical patients, many of whom are at a high risk of aspiration. Instrumental assessment of swallowing is critically important and service delivery for this at-risk patient population was significantly impacted by the pandemic. In many institutions, instrumental assessment was halted or eliminated from the clinical workflow at the beginning of the pandemic, leaving clinicians without evidence-based gold standards to definitively evaluate swallowing function. This study describes a successful return to best practice in swallowing assessment in the context of the COVID-19 pandemic.

**Abstract:**

The aim of this study is to describe a measured return to instrumental dysphagia assessments for our vulnerable surgical patient population, such that best practice patterns could be resumed and our staff kept safe from transmission of COVID-19. A retrospective medical record review provided data on clinical practice patterns of swallowing assessment in an at-risk surgical patient population. Outcomes of this study support protocols that allow clinicians to safely resume the use of instrumental assessment and return to best practice in dysphagia assessment for our surgical patient population.

## 1. Introduction

Normal swallowing function is a complex process, which allows for the safe transfer of food and liquid to the stomach while providing protection for the airway [1,2,3]. Disorders of swallowing, including evidence of aspiration, if undiagnosed, may lead to medical complications such as aspiration pneumonia, malnutrition and dehydration, may extend the duration of hospitalizations and increase the overall cost per hospital admission, or in the worst of cases, result in increased morbidity and mortality [1,2,3,4,5,6,7,8,9]. Treatment plans for disorders of swallowing can be developed after instrumental, objective assessment is conducted. Instrumental assessment allows the provider to determine the degree and nature of impairment and develop a plan for intervention [1].

COVID-19 has challenged our use of best clinical practices. The two instrumental assessments that are widely considered to be the gold standard for evaluating swallowing function are the Modified Barium Swallow (MBS) study and Fiberoptic Endoscopic Evaluation of Swallowing (FEES) [6,10]. MBS studies are conducted by trained providers within the radiological suite under video fluoroscopy, during which dynamic moving images of the swallowing mechanism are recorded to evaluate swallowing function. During FEES studies, clinicians perform naso-endoscopy to capture the dynamic mechanism of swallowing, which also allows for the direct visualization of the vocal folds to rule out laryngeal pathology [1]. COVID-19 challenged these practices from a staff safety perspective, as practitioners are required to be in close proximity to the unmasked patient during these assessments. Additionally, coughing is an inherent part of instrumental assessment, either due to spontaneous generation or via instruction from the clinician or physician provider. It was determined early in the pandemic that MBS and FEES were considered to be procedures with a higher risk category for COVID-19 transmission [11,12,13].

Accurate assessment of swallowing in surgical patients was especially challenging, as some surgical patients are at high risk from complications of dysphagia. Surgical patients that have been identified in the literature as high risk for post-operative dysphagia include those undergoing cervical spine surgery, cardio-thoracic surgery, thyroid surgery, laryngeal and pulmonary surgeries, esophageal surgery, gastric surgery, anti-reflux surgery, neurological and cortical surgeries, as well as surgeries involving the cranial nerves [3,14].

The literature has identified important factors related to dysphagia post-surgery. There are both shared factors that are associated with dysphagia in the surgical patient population, as well as factors that vary based on the type of surgical procedure. For example, in the cardiac surgical patient population, factors associated with dysphagia include advanced age, intubation time, surgical conditions and the presence of comorbidities such as diabetes, renal insufficiency, hyperlipidemia and pre-operative congestive heart failure, as well as in those patients undergoing non-coronary artery bypass procedures [4,5,9]. In spinal surgeries, increased age, increased operative time, multilevel surgery, revision surgery, cervical level injuries, plate fixation, plate prominence, severe pain, tracheostomy placement and female gender are found as being associated with increased risk of post-surgical dysphagia [10,15,16,17,18]. Rates of post-surgery dysphagia vary widely in the literature, from 1% to as high as 81%, depending upon the type of surgical intervention [1,2,4,8,15,16,18,19,20,21].

Instrumental swallowing assessment is particularly important in patient populations that are at high risk for dysphagia. Not only does instrumental assessment provide definitive information about the type and severity of the dysphagia, but it also provides critical information about bolus flow within the pharynx. The results of instrumental assessment inform the clinician on how to best treat the dysphagia and guide recommendations for oral diet consumption. This important clinical information can be gleaned from both MBS and FEES, though surgical patients often benefit from FEES as it is portable and can be completed at the patient’s bedside without the need to transport medically complex patients to the radiology suite as is the case with MBS studies. FEES also provides the ability to view the larynx and assess the integrity of the vocal folds, which protect the airway during the swallow. This is an important consideration in the surgical population, as there is risk of damage to the vocal folds during intubation and/or surgical procedures [1].

At the onset of the pandemic, published literature provided guidance for the use of instrumental dysphagia assessment that differed significantly from the standard of care that clinicians were accustomed to delivering. Concern for staff safety in the context of COVID-19 transmission was evident in studies that provided information on recommended personal protective equipment [22], prioritization of FEES for only the most critically ill [23] and recommendations for the use of non-instrumental assessments [24]. Based on the available science and out of concern for staff safety, our facility, like many other healthcare institutions, chose to discontinue instrumental swallowing diagnostic procedures, including FEES and MBS, during the initial stages of the pandemic. This was especially problematic for surgical patients due to the increased risk for dysphagia in this population. During this time, a shift occurred in which clinicians were returning to non-instrumental means of assessing swallowing. While this clinical practice supported keeping staff safe, it was not consistent with best practice in swallowing assessment. In our institution, it was the desire to return to best practice that drove the implementation of protocols that allowed for an early return the use of instrumentation for swallowing assessment for our vulnerable surgical patient population [25].

The purpose of this study was to describe a successful measured return to evidence-based best practices in swallowing assessment of surgical patients at our large, urban tertiary care center, and to determine if re-implementing the use of the clinical gold standard for dysphagia assessment, FEES, was safe for our staff during the COVID-19 pandemic.

## 2. Materials and Methods

### 2.1. Patient Population

Participants for this retrospective study were hospitalized patients over the age of 18 in our tertiary care facility who underwent a surgical procedure during their hospital stay and for whom a swallowing consult had been placed by their medical provider.

### 2.2. Procedure

This study was conducted as a retrospective medical record review and was approved by both the Yale School of Medicine and Southern Connecticut State University’s Institutional Review Board. Data extraction took place from the electronic medical record of a period of time between 1 March 2020 and 6 October 2021. The following data points were extracted from the medical record: patient demographics; type and date of surgery performed; date of swallow evaluation consult and evaluation; type of swallowing assessment performed (FEES, MBS, Clinical Swallowing Evaluation (CSE), swallow screening); and COVID-19 status. A positive COVID-19 encounter was defined by the institution as an encounter in which: 1. The patient had a positive COVID-19 lab result during hospitalization; OR 2. The patient had a positive COVID-19 lab up to 7 days before admission AND did not have a negative result between the positive result and admission. In addition to the extracted data, information on incidence of COVID-19 positive providers and information on contact tracing were extracted from departmental statistics. Frequency counts were used to summarize data and provide descriptive statistics of study outcomes. A chi-square analysis of independence was performed to examine the relationship between timing of instrumental assessment and risk of surgical procedure as it relates to dysphagia.

### 2.3. Clinical Protocols and Algorithms

Clinical protocols, while not a focus of this study, are relevant to the methodology in that the return to the use of instrumental assessment was measured and altered from pre-pandemic workflow. A brief discussion outlines this critical information regarding clinical practice during this time. Importantly, personal protective equipment (PPE) was secured early in the pandemic and used universally with our staff. PPE during the study period included N-95 masks, gloves and gowns for the entire period. Eye protection was added as a requirement and is currently used. Head and foot coverings were optional at our institution. COVID-19 testing protocols for staff were conducted per institutional guidelines.

Clinical algorithms for service delivery were developed during this timeframe, and the maintenance of workflow practices was a dynamic process, evolving as information about the virus became more readily available. Early in the pandemic, instrumental assessments were not being utilized in alignment with service delivery in Otolaryngology clinics at our institution. As the pandemic progressed, instrumentation resumed, but in a measured fashion. Algorithms were established that guided prioritization of patients eligible for instrumental assessment, necessary COVID-19 testing for patients and proper PPE for staff. Over the course of the study period, these algorithms shifted toward a more liberal use of instrumentation, more autonomy of staff in decision making and less stringent COVID-19 testing procedures for patients [25].

## 3. Results

### 3.1. Study Sample

The surgical patient extraction sample consisted of 1849 patient hospitalizations who were admitted to our tertiary care facility and underwent at least one surgical procedure during the study period. The study sample was heterogeneous and representative of a typical inpatient population, in that patients from all service lines were included in the study. The distribution of patients across service lines can be found in Table 1. Internal Medicine had the largest number of hospitalizations (340), followed by Cardiothoracic Surgery (225), Hospitalist (177), Neurosurgery (172), Otolaryngology (131), Surgery (95), Cervical Spine (87), Medical ICU (77) and Stroke (77).

### 3.2. Surgical Demographics

The number and types of surgical procedures performed during the study period can be found in Table 2. In this analysis, surgical procedures were categorized based on surgical specialty and clinical relevance to this study. The following surgical specialty categories were analyzed due to their known association with dysphagia: cardiac; gastroenterology; neurosurgery; cervical spine; and otolaryngology. A general category was created to include those surgeries that did not fall into the high-risk category. Of the categories known to be associated with dysphagia, gastroenterology had the highest prevalence (255 procedures), followed by neurosurgery (182), cardiac (167) and otolaryngology (164). Cervical spine surgery was found to be the least prevalent, with 52 surgical procedures.

### 3.3. Swallowing Consults and Evaluations

The surgical patient extraction sample consisted of 3428 consults placed for swallowing evaluations over 1849 hospitalizations. Of these hospitalizations, 1093 hospitalizations had one or more Fiberoptic Endoscopic Evaluation of Swallowing (FEES) performed, 1175 hospitalizations had one or more YSP (Yale Swallow Protocol), 138 had one or more Clinical Swallow Evaluation (CSE) and 182 had one or more Modified Barium Swallow (MBS) (Table 3). The most frequently performed swallowing assessment was FEES (1974 evaluations), followed by YSP (1337 screens), MBS (232 evaluations), and lastly CSE (174 evaluations).

### 3.4. COVID-19 Status and Swallowing Evaluations

One hundred sixteen hospitalizations that had swallowing consults placed were deemed to be COVID-19 positive by the institution. In 48 of these hospitalizations, the primary diagnosis was COVID-19. During these hospitalizations, 141 FEES were completed (over 78 hospitalizations), as well as 73 YSP (over 58 hospitalizations), 8 CSE (11 hospitalizations) and 8 MBS (8 hospitalizations) (Table 4a). The distribution of COVID-19 positive surgeries can be found in Table 4b.

### 3.5. Timing of Instrumental Evaluations

The timing of instrumental evaluations (FEES and MBS) was investigated using a sub-set of the data that consisted of hospitalizations with surgical procedures that occurred on a single day (*n* = 1300). Hospitalizations with multiple surgical dates were excluded from this analysis. Two hundred and fifty hospitalizations had instrumental evaluations that occurred before the surgery (376 total evaluations), 632 hospitalizations had instrumental evaluations that took place after the surgery (1001 total evaluations) and only 20 hospitalizations had instrumental evaluations that took place on the same day as the surgery (20 total evaluations) (Table 5).

These data were further analyzed by surgical specialty (Table 6). A chi-square test of independence showed an association between timing of swallowing evaluations and risk of associated dysphagia [*X*^2^ (2, *n* = 1377)] = 248.05, *p* < 0.000001. The neurosurgery, cardiac, otolaryngology and cervical spine categories demonstrated discrepancies between pre- and post-surgical instrumental assessments, with more swallowing assessments being performed in the post-operative time period than before surgery. Gastroenterology and general surgery had a more even distribution of frequencies of pre- and post-surgical instrumental assessments.

### 3.6. Staff Outcomes and Contact Tracing Data

Departmental data revealed that no inpatient SLP staff member who performed instrumental swallowing evaluations had a COVID-19 positive test result related to workplace exposure during the study period. Additionally, the Speech-Language Pathology department was not notified by Infection Prevention with concern of COVID-19 spread due to the use of instrumentation or to determine contract tracing data for SLP staff during the study period.

## 4. Discussion

COVID-19 has altered the delivery of healthcare worldwide. During the initial phase of the pandemic, many best practice procedures were halted out of concern for staff safety in the context of workforce preservation. In the field of Speech-Language Pathology, the pandemic challenged the use of best practice evaluation methods to assess swallowing safety. In March 2020, the use of instrumental assessment for swallowing ceased at our institution while administration gathered information about SARS-CoV-2 virus and how to protect both staff and patients when performing procedures that placed staff at high risk for transmission of COVID-19. This workforce-driven decision left patients without access to best practices in swallowing assessment, which was particularly challenging for our vulnerable surgical patient population. The present study describes a measured return to instrumental dysphagia assessments for this patient population such that best practice patterns could be resumed and staff be kept safe from transmission of COVID-19.

The use of instrumentation for swallow evaluations is particularly important with critically ill hospitalized patients. These assessments are part of a clinical algorithm that allows SLPs to definitively diagnose and subsequently treat dysphagia. Without the use of evidence-based practice in swallowing assessment, the diagnosis of dysphagia can be missed due to silent aspiration, whereby food or liquid passes into the trachea without overt signs of aspiration such as coughing or choking. The inability to accurately diagnose dysphagia can lead to negative sequelae, such as aspiration pneumonia, which can lead to longer hospitalizations and less favorable clinical outcomes.

Despite initial shifts in service delivery, an early return to the use of instrumental swallowing studies was critical to resuming best practice for dysphagia assessment. In our institution, best practice patterns dictate the completion of an initial swallow screen. The Yale Swallow Protocol (YSP) is a validated screening tool that has been shown to be effective in screening hospitalized patients for aspiration risk [26]. If the YSP is passed, the patient can be placed on a diet. If failed, they undergo an instrumental assessment. The instrumental evaluation is critical at this point in the clinical pathway. Without it, dysphagia cannot be effectively diagnosed and managed, leading to less favorable patient outcomes.

While practice patterns shifted during this time period, results of this study support that swallowing evaluations remained an important part of clinical workflow, and a return to instrumental assessment was a critical component of the evidence-based practice paradigm. Results from this study demonstrate that FEES was the predominant evaluation type (1974 evaluations) (Table 3). This is in keeping with clinical workflow at our institution, and demonstrates that despite initial cessation of instrumental assessments, a robust number of patients received FEES during a time when the literature did not yet support resuming instrumentation.

Surgical patients can be particularly susceptible to swallowing difficulties. General factors like intubation time during the procedure [4,9] and deconditioning during hospitalization and recovery [1,8] can contribute to the likelihood of post-operative dysphagia. Additionally, certain surgical procedures that directly impact the anatomy and physiology of the swallowing mechanism are associated with higher prevalence of post-operative dysphagia [10,15,16,17,18]. The inability to accurately diagnose dysphagia in the surgical patient population can have significant implications for surgical recovery. Dysphagia can lead to negative consequences, including but not limited to inadequate nutrition, poor wound healing and other negative respiratory sequelae, which can contribute to post-operative complications.

The surgical patient population shifted significantly at our institution during the pandemic, in that, during the initial months, only necessary surgical procedures were completed. Elective procedures diminished significantly during this time. As a result, our study population represents patients who underwent urgent or emergent surgery during the pandemic. Institutional policy dictated implementation of COVID-19 surgical protocols for COVID-19 positive patients. COVID-19 positive status or active infection was not a deciding factor in surgical decision making, though it initiated a set of protocols to ensure the safety of the surgical team. Throughout the study period, data support that a significant need for evidence-based swallowing evaluation remained. While clinical practice workflow was challenged by the initial cessation of instrumental swallowing evaluations, administration at our institution prioritized return to evidence-based practice using instrumentation early in the months that followed the initial shut-down [25]. Initial measures to reduce the spread of COVID-19 and preserve the clinical workforce included pre-procedure COVID-19 testing, as well as administrative approval for instrumental assessments on a case-by-case basis. As the pandemic progressed, these measures were re-evaluated on a regular basis to align with Otolaryngology and Infection Prevention, and instrumentation practice was gradually expanded to a more robust use of instrumental assessment and resumption of best practice in swallowing assessment.

During the study period, 3428 consults were placed for swallowing evaluations on patients who had surgery over 1849 hospitalizations. Patients who underwent surgical procedures were admitted under a variety of service lines within our large, urban tertiary care facility. Internal Medicine had the largest number of hospitalizations that had consults placed for swallowing evaluations (340), followed by Cardiothoracic Surgery (225), Hospitalist (177), Neurosurgery (172), Otolaryngology (131), Surgery (95), Cervical Spine (87), Medical ICU (77) and Stroke (77) (Table 1). It is important to note that the distribution of patients who had surgery for whom swallow consults were placed had likely shifted due to the pandemic. It might be expected that patients who had surgery would be more typically represented in the surgical services, however due to a significant decline in elective or non-urgent surgeries during this time, the majority of patients who had surgeries were instead admitted to medical services (Internal Medicine and Hospitalist Service). These were patients with medical diagnoses that required surgical consultation versus patients that came into the hospital with a primary surgical need.

Re-analyzing these data by surgical specialties instead of service provides additional insight to the surgical patient population during this timeframe (Table 2). For the purposes of analysis, surgical specialties were categorized based on procedures that have a known association with a diagnosis of dysphagia. Of these categories, gastroenterology had the highest number of procedures (255), followed by neurosurgery (182), cardiac (167), otolaryngology (164) and cervical spine (52). General surgery was characterized by procedures that do not have an association with a high risk of dysphagia. This analysis allows for a more detailed understanding of the distribution of surgical procedures during the pandemic when only the most critical procedures were being performed.

The timing of the instrumental assessment in relation to the surgical date is of interest in this clinical population. In the sub-set of patients who had surgery on a single day (*n* = 1397) of the hospitalization, 71% (1001/1397) of instrumental swallowing evaluations were completed after the surgery took place, while 27% (376/1397) took place before and only 1% (20/1397) of instrumental evaluations took place on the same day of surgery (Table 5). The fact that the majority of the instrumental evaluations were completed post-operatively in this patient population is not unexpected, however the number of pre-operative swallowing evaluations is higher than one might expect when considering a population with surgery as a primary diagnosis. This is likely due to the fact that due to COVID-19, the number of true surgical patients was significantly diminished compared to the pre-pandemic period. Many of the patients who underwent surgery were on non-surgical services, and in fact, a more heterogeneous patient population who underwent surgery, suggesting that other co-morbidities could be contributing to the need for pre-operative instrumental swallowing evaluations. Further investigation of the factors driving these data would be of interest in future studies.

This hypothesis is further supported by considering the timing of instrumental swallowing evaluations in the context of surgical categories (Table 6). Analysis of these data reveals that the vast majority of the surgical specialty areas whose procedures have an association with dysphagia had significantly higher volumes of instrumental swallowing assessments on patients post-operatively than were completed on patients pre-operatively or on the same day. This assessment pattern was seen for the surgical categories of neurosurgery, cardiac, otolaryngology and cervical spine. These data corroborate what is known about these high-risk procedures in that in the majority of the cases, concerns for swallowing function arose in the post-operative period.

The referral pattern for gastroenterology was more evenly distributed regarding timing of swallowing evaluations. We hypothesize that the reason for this is multi-factorial. In terms of risk, the category of gastroenterology is categorized as a moderate risk given that is a hybrid category, in that it includes surgical procedures that are both associated and not associated with oropharyngeal dysphagia. In this way, the category of gastroenterology would not be considered high risk for the purposes of this study, and thus we would not expect to see the same pattern in terms of timing of evaluation. Additionally, we would expect referral patterns to be different for gastroenterology, given that many of these patients initially present with complaints of swallowing difficulty and that instrumental assessment is often a critical component of the diagnostic process. These factors may have contributed to the more even distribution of instrumental assessments pre- and post-operatively. Finally, the general category also reveals similar distribution, reflecting referral patterns as would be expected, given no presumed causal relationship between those lower-risk surgeries and dysphagia.

Data demonstrate that despite the concerns of staff safety during the pandemic, instrumental assessment for swallowing evaluations in our surgical patient population remained a critical need. In order to keep staff safe, clinical algorithms were put in place early in the pandemic in an attempt to resume best practice for patients and to preserve the workforce in the context of concern for transmission of COVID-19. These protocols were initially somewhat limiting in terms of swallowing instrumentation as administration worked to keep staff safe. Over time, protocols were altered in keeping with institutional and departmental changes to eliminate the necessity for pre-procedure COVID-19 testing, as well as the administrative approval for use of instrumentation. These changes allowed clinicians to resume more autonomy in clinical decision making, and resulted in increased patient access to instrumental assessment. It was found that eliminating these early precautionary measures did not change staff contact tracing data, and ultimately resulted in staff remaining safe through this period. The current algorithm remains as it was in place toward the end of the study period, and involves clinical decision making in the context of the COVID-19 status of the patient in real time. Active infection is determined by Infection Prevention and precautions are put in place. This information allows SLPs to engage in risk assessment on a case-by-case basis, and informs decision making such that SLPs are able to provide appropriate and critical care necessary to the health of the patient while minimizing staff exposure.

### Limitations and Future Directions

The study design results in an inherent limitation of any research investigating a particular timeframe that requires deviation from normal practice patterns. Results of this study reflect practice patterns during the COVID-19 time period only, and therefore results may not be generalizable to the post-pandemic period in that distributions of patients by service and timing of assessments may have been influenced by the admissions, workflow and consult pattern of the pandemic. Additionally, despite the robust number of referrals and instrumentals that took place during this timeframe, the number of COVID-19 positive encounters was relatively low compared to non-COVID-19 encounters. A multi-site design may be considered to increase the sample size in future investigations.

This study can serve as an important foundation for future studies, whereby trends in the assessment of swallowing function in surgical patients can be considered. A replication study in the current post-pandemic timeframe could provide additional insight into the factors contributing to these data. Future investigations involving surgical patients may consider additional factors like age and dysphagia outcomes, as well COVID-19 status, to further guide clinical practice in this patient population.

## 5. Conclusions

The SARS-CoV-2 virus altered clinical practice patterns worldwide. Clinical directives in the literature early in the pandemic dictated the use of non-instrumental assessment for the evaluation of swallowing function. The initial inability to use best practice gold standards for dysphagia evaluation challenged clinical practice in Speech-Language Pathology, particularly for at-risk surgical patients. Results from this study support that the use of instrumental swallowing assessment remained a critical need during the pandemic. Findings support the field resuming instrumental assessment of this vulnerable patient population, and demonstrate that a measured return to the use of instrumental assessment can be successful in providing best practice swallowing assessment to this at-risk surgical patient population while keeping staff safe during the COVID-19 pandemic.

## Figures and Tables

**Table 1:** Primary Patient Service Line.

Service Line	Number of Hospitalizations
Internal Medicine	340
Cardiothoracic Surgery	225
Hospitalist Service	177
Neurosurgery	172
Otolaryngology/ENT	131
Surgery	95
Cervical Spine	87
Medical ICU	77
Stroke	77
General Medicine	67
Neurosurgery	61
Oncology	48
Cardiology	37
Surgical ICU	27
Thoracic	27
Trauma	25
Neuro ICU	19
Vascular Surgery	15
Transplant	14
Emergency General Surgery	11
Hematology	11
Neurosurgery Stroke Inpatient Team	11

Primary service line with 10 or fewer hospitalizations: Acute Pain Service; Bariatric Surgery; Cardiac ICU; Electrophysiology; Emergency Medicine; Endocrine Surgery; Gynecology; Hepatology; Infectious Disease; Medical I-Team; Neonatal ICU; Nephrology; Neurosurgery General Inpatient Team; Neuro-Oncology; Palliative Care; Pediatric Hematology/Oncology; Plastic Surgery; Podiatry; Pulmonology; Urology; and Virtual Hospice.

**Table 2:** Surgical Episodes.

Surgical Category	Surgical Episodes	Percent of Total Episodes
General	478	36.8
Gastroenterology	255	19.6
Neurosurgery	182	14.0
Cardiac	167	12.9
Otolaryngology (ENT)	164	12.6
Cervical Spine	52	4.0
Total	1298	

**Table 3:** Swallow Evaluation Type.

Evaluation Type	Hospitalizations with at Least One Swallow Evaluation *	Evaluations Completed *
Flexible Endoscopic Evaluation of Swallowing (FEES)	1093 (42.2)	1974 (53.1)
Modified Barium Swallow (MBS)	182 (7.0)	232 (6.2)
Clinical Swallow Evaluation (CSE)	138 (5.3)	174 (4.7)
Yale Swallow Protocol (YSP)	1175 (45.4)	1337 (36.0)
Total	2588	3717

* Percentage of column total in parentheses.

**Table 4:** (a) COVID-19 Demographics. (b) COVID-19 Surgical Distribution by Category.

(a)		
**Evaluation Type**	**COVID-19 Positive Hospitalizations with One or More Swallow** Evaluation *	**Evaluations** Completed *
Flexible Endoscopic Evaluation of Swallowing (FEES)	78 (51.3)	141 (60.5)
Modified Barium Swallow (MBS)	8 (5.3)	8 (3.4)
Clinical Swallow Evaluation (CSE)	8 (5.3)	11(4.7)
Yale Swallow Protocol (YSP)	58 (38.2)	73 (31.3)
Total	152	233
(b)		
**Surgical Category**		**Percent of Total COVID-19 Positive** **Encounters**
General		42.8
Gastroenterology		22.9
Otolaryngology (ENT)		20.9
Neurosurgery		6.7
Cardiac		3.8
Cervical Spine		2.9

* Percentage of column total in parentheses.

**Table 5:** Timing of Instrumental Examinations.

Timing of Instrumental Exam	Total Hospitalizations with Instrumentals *	Total Instrumental Evaluations *
Before surgery	250 (27.7)	376 (26.9)
Day of surgery	20 (2.2)	20 (1.4)
After surgery	632 (70.1)	1001 (71.7)
Total	902	1397

* Percentage of column total in parentheses.

**Table 6:** Timing of Instrumental Examinations by Surgical Specialty.

Surgical Category	Instrumental Examinations before Surgery *	Instrumental Examinations Day of Surgery *	Instrumental Examinations after Surgery *	Associated Risk for Dysphagia
General	214 (56.9)	11 (55.0)	250 (25.0)	Low
Gastroenterology	101 (26.9)	2 (10.0)	112 (11.2)	Moderate
Neurosurgery	33 (8.8)	3 (15.0)	180 (18.0)	High
Cardiac	11(2.9)	3 (15.0)	228 (22.8)	High
Otolaryngology (ENT)	14 (3.7)	1 (5.0)	191 (19.1)	High
Cervical Spine	3 (0.8)	0	40 (4.0)	High
Total	376	20	1001	

* Percentage of column total in parentheses.

## Data Availability

The data presented in this study are available on request from the corresponding author. The data are not publicly available due to privacy of the medical health record.

## References

[1] Black R.J., Novakovic D., Plit M., Miles A., MacDonald P., Madill C. (2021). Swallowing and laryngeal complications in lung and heart transplantation: Etiologies and diagnosis. J. Heart Lung Transplant..

[2] Grimm J.C., Magruder J.T., Ohkuma R., Dungan S.P., Hayes A., Vose A.K., Orlando M., Sussman M.S., Cameron D.E., Whitman G.J. (2015). A novel risk score to predict dysphagia after cardiac surgery procedures. Ann. Thorac. Surg..

[3] Vansant M.B., Kunduk M., McWhorter A.J. (2016). A review of postsurgical dysphagia in nonmalignant disease. Curr. Opin. Otolaryngol. Head Neck Surg..

[4] Barker J., Martino R., Reichardt B., Hickey E.J., Ralph-Edwards A. (2009). Incidence and impact of dysphagia in patients receiving prolonged endotracheal intubation after cardiac surgery. Can. J. Surg..

[5] Ferraris V.A., Ferraris S.P., Moritz D.M., Welch S. (2001). Oropharyngeal dysphagia after cardiac operations. Ann. Thorac. Surg..

[6] Love A.L., Cornwell P.L., Whitehouse S.L. (2013). Oropharyngeal dysphagia in an elderly post-operative hip fracture population: A prospective cohort study. Age Ageing.

[7] Marciano R.D., Seaman B., Sharma S., Wood T., Karas C., Narayan K. (2018). Incidence of dysphagia after odontoid screw fixation of type II odontoid fracture in the elderly. Surg. Neurosurg..

[8] Plowman E.K., Anderson A., York J.D., DiBiase L., Vasilopoulos T., Arnaoutakis G., Beaver T., Martin T., Jeng E.I. (2021). Dysphagia after cardiac surgery: Prevalence, risk factors, and associated outcomes. J. Thorac. Cardiovasc. Surg..

[9] Werle R.W., Steidl E.M.D.S., Mancopes R. (2016). Oropharyngeal dysphagia and related factors in post-cardiac surgery: A systematic review. CoDAS Soc. Bras. Fonoaudiol..

[10] Smith-Hammond C.A., New K.C., Pietrobon R., Curtis D.J., Scharver C.H., Turner D.A. (2004). Prospective analysis of incidence and risk factors of dysphagia in spine surgery patients: Comparison of anterior cervical, posterior cervical, and lumbar procedures. Spine.

[11] Muraleedharan M., Kaur S., Arora K., Singh Virk R.S. (2020). Otolaryngology practice in COVID 19 Era: A road-map to safe endoscopies. Indian J. Otolaryngol. Head Neck Surg..

[12] Guda N.M., Emura F., Reddy D.N., Rey J., Seo D., Gyokeres T., Tajiri H., Faigel D. (2020). Recommendations for the operation of endoscopy centers in the setting of the COVID-19 pandemic—World Endoscopy Organization guidance document. Dig. Endosc..

[13] Namasivayam-MacDonald A.M., Riquelme L.F. (2020). Speech-language pathology management for adults with COVID-19 in the acute hospital setting: Initial recommendations to guide clinical practice. Am. J. Speech-Lang. Pathol..

[14] Tsuboi K., Lee T.H., Legner A., Yano F., Dworak T., Mittal S.K. (2011). Identification of risk factors for postoperative dysphagia after primary anti-reflux surgery. Surg. Endosc..

[15] Miles A., Jamieson G., Shasha L., Davis K. (2021). Characterizing dysphagia after spinal surgery. J. Spinal Cord. Med..

[16] Radcliff K.E., Koyonos L., Clyde C., Sidhu G.S., Fickes M., Hilibrand A.S., Albert T.J., Vaccaro A.R., Rihn J.A. (2013). What is the incidence of dysphagia after posterior cervical surgery?. Spine.

[17] Riley L.H., Vaccaro A.R., Dettori J.R., Hashimoto R. (2010). Postoperative dysphagia in anterior cervical spine surgery. Spine.

[18] Segebarth B., Datta J.C., Darden B., Janssen M.E., Murrey D.B., Rhyne A., Beckham R., Ponce C. (2010). Incidence of dysphagia comparing cervical arthroplasty and ACDF. SAS J..

[19] Kalb S., Reis M.T., Cowperthwaite M.C., Fox D.J., Lefevre R., Theodore N., Papadopoulos S.M., Sonntag V.K. (2012). Dysphagia after anterior cervical spine surgery: Incidence and risk factors. World Neurosurg..

[20] Martin R.E., Neary M.A., Diamant N.E. (1997). Dysphagia following anterior cervical spine surgery. Dysphagia.

[21] Reinard K.A., Cook D.M., Zakaria H.M., Basheer A.M., Chang V.W., Abdulhak M.M. (2016). A cohort study of the morbidity of combined anterior-posterior cervical spinal fusions: Incidence and predictors of postoperative dysphagia. Eur. Spine J..

[22] Lammers M.J.W., Lea J., Westerberg B.D. (2020). Guidance for otolaryngology health care workers performing aerosol generating medical procedures during the COVID-19 pandemic. J. Otolaryngol. Head Neck Surg..

[23] Frajkova Z., Tedla M., Tedlova E., Suchankova M., Geneid A. (2020). Postintubation dysphagia during COVID-19 outbreak-contemporary review. Dysphagia.

[24] Brodsky M.B., Gilbert R.J. (2020). The long-term effects of COVID-19 on dysphagia evaluation and treatment. Arch. Phys. Med. Rehabil..

[25] Warner H., Young N. (2023). Best Practice in Swallowing Assessment in COVID-19. Dysphagia.

[26] Leder S.B., Suiter D.M. (2014). The Yale Swallow Protocol. An Evidence-Based Approach to Decision Making.

